# Fibrodysplasia ossificans progressiva complicated with post traumatic and infectious myositis ossificans in masseter: A case report

**DOI:** 10.1097/MD.0000000000039648

**Published:** 2024-09-13

**Authors:** Yian Guan, Dongyang Ma

**Affiliations:** a Department of Orthodontics, School of Stomatology, Lanzhou University, Lanzhou, China; b Department of Oral and Maxillofacial Surgery, the 940th Hospital of Joint Logistics Support Force of PLA, Lanzhou, China.

**Keywords:** Fibrodysplasia ossificans progressive, Heterotopic ossification, Masseter, Myositis ossificans

## Abstract

**Rationale:**

Myositis ossificans (MO) is characterized by benign heterotopic ossificans in soft tissues like muscles, which can be classified into nonhereditary MO and fibrodysplasia ossificans progressiva (FOP). Nonhereditary MO is characterized by ossification of the soft tissues after acute or repetitive trauma, burns, or surgical intervention. FOP is a rare and crippling disease characterized by congenital malformation of the big toe and heterotopic ossification in muscle. The majority of FOP’s musculoskeletal traits are associated with dysregulated chondrogenesis. The diagnosis is mainly based on clinical manifestation, imaging examination, and genetic analysis. There is still no effective treatment to cure or slow its progression. The best approach remains early diagnosis, conservative drug treatment, and injury prevention to avoid local ossification.

**Patient concerns:**

A 34-year-old male presented at our hospital because of trismus caused by ossification of the masseter muscle. In addition, he had serious stiffness and multiple bony masses throughout the body, which led to limited movement.

**Diagnoses:**

Based on the clinical manifestation of movement restriction, characteristic radiographic images of ossification of soft tissues, the genetic test showing a heterozygous molecule (c.974G > C, p.G325A) of the activin A receptor type I, the patient was diagnosed as FOP complicated with localized MO in masseter after trauma and infection.

**Interventions:**

The patient underwent the surgical resection of ossification in the masseter muscle, he was instructed to insist on mouth-opening exercises and take glucocorticoids and nonsteroidal anti-inflammatory medications after surgery.

**Outcomes:**

The symptoms of trismus are relieved, and eating can be basically achieved after surgery, while the symptoms of trismus recurred 2 years later.

**Lessons:**

Although FOP has unique clinical manifestations, its diagnosis may be difficult because of its rarity. Gene analysis is the main standard for diagnosis, while patients with different genotypic variations may show different clinical symptoms. Therapeutic interventions are still supportive and preventive, and surgery is not recommended except under certain circumstances.

## 1. Introduction

Myositis ossificans (MO) is defined by benign heterotopic ossificans in soft tissues such as muscles, which can be divided into 2 types: nonhereditary MO and fibrodysplasia ossificans progressiva (FOP).^[1]^ Nonhereditary MO is defined as the ossification of soft tissues following acute or recurrent trauma, burns, or surgical intervention.^[2]^ FOP is a rare genetic disorder of bone morphogenetic protein (BMP) characterized by multiple ectopic ossifications in the whole body.^[3]^ Most patients with FOP have the same recurrent single nucleotide change in activin A receptor type I (ACVR1), a BMP type I receptor subtype.^[4]^ The classical molecule (c.617G > A, p.R206H) is usually accompanied by typical clinical features of FOP, including valgus deformity of the great toes and extensive heterotopic ossification at a young age.^[5]^

This paper reports a man carries a unique, heterozygous, missense molecule (c.974G > C, p.G325A), which may lead to his rare clinical symptoms.

## 2. Case report

A 34-year-old male presented at our hospital because of the inability to open his mouth for 1 and a half years after the incision and drainage of the right masseter space abscess.

### 2.1. Medical history

The patient had numbness and pain in the lower limb and gradually developed a severely limited range of motion and rigidity of that hip at about 21 years old. In the following 5 years, his lumbar vertebrae had swelling with mild pain, and limitation in range of motion progressed, then he was also diagnosed with ankylosing spondylitis. At the age of 26, he was suspected of having bilateral rib fractures by a local doctor after a car accident, but no imaging examination or further treatment was performed. After the injury, he tried to reduce the activity of his upper limbs because of pain, but after a month, he found that he could not lift his left arm gradually. Then, he had stiffness of the neck, and moving was limited due to a lack of neck extension at the age of 28. At the age of 32, due to the pain in the right mandibular wisdom teeth and following facial swelling for more than 1 month, he was diagnosed with right masseter space infection and marginal osteomyelitis of the mandible caused by dental abscesses in the local hospital. The patient underwent incision and drainage of a masseter space abscess under general anesthesia. After the operation, facial pain was alleviated, but the limitation of mouth opening was gradually aggravated and progressed to trismus. About half a year later, he was admitted to our hospital due to difficulty in eating. Besides, the patient stated that there are no similar symptoms or illnesses among his family members.

### 2.2. Clinical examination

**Figure 1.**
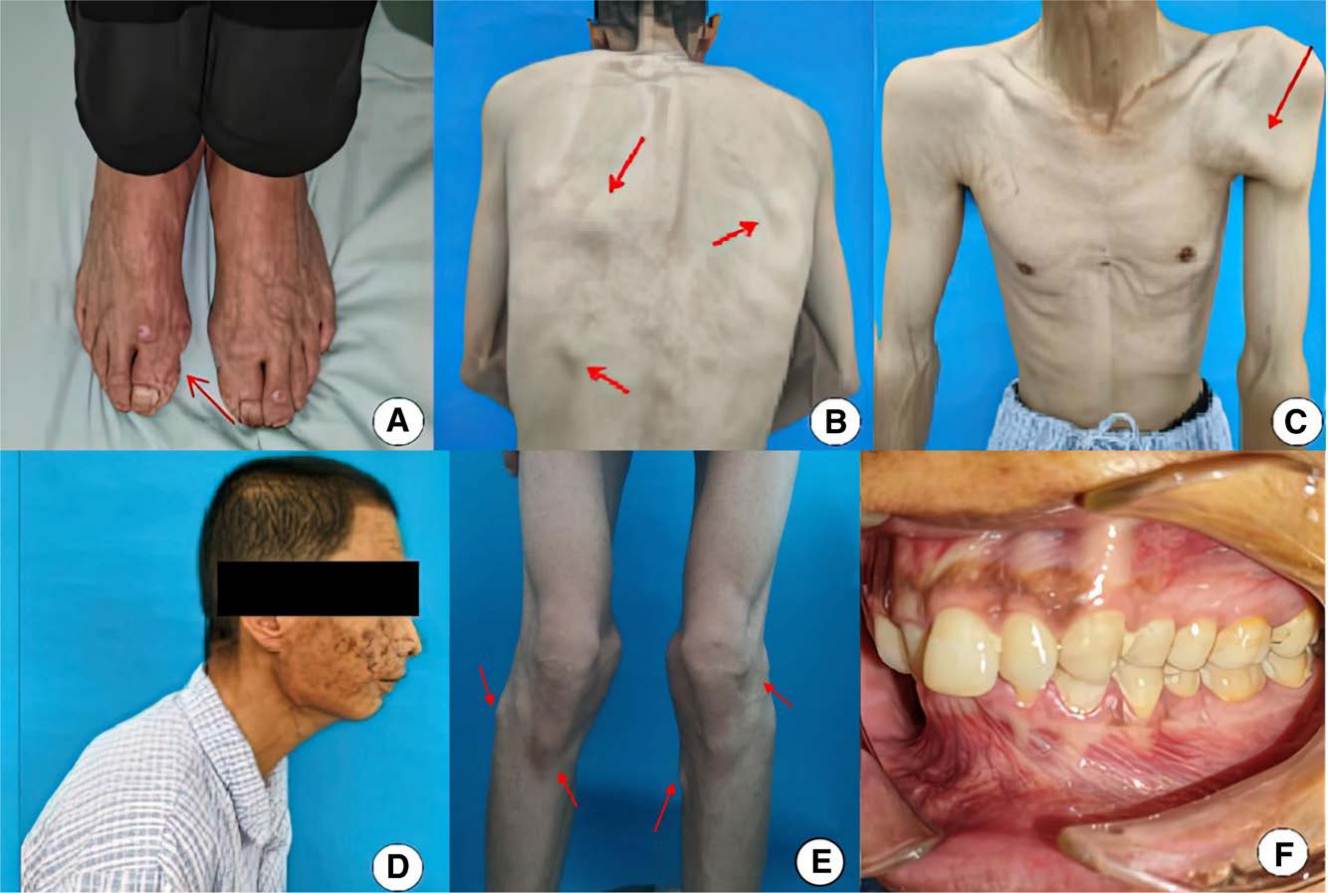
Preoperative images. (A) The symptom of congenital thumb deformity was not obvious; (B) Palpable masses in the shoulder and back region; (C) A hard cord in the left axilla; (D) Overbite appearance and spinal deformity of kyphosis; (E) Presence of masses on the knee; (F) Posterior occlusion.

The patient was emaciated due to eating disorders, he was not able to walk and squat in normal posture due to a spinal deformity of kyphosis, he also had a restricted range of motion of the left arm, and there were multiple palpable nodules on his shoulder, back, and knee region, and a cord-like mass was seen on the left axilla. Facial asymmetry was observed, with the right side slightly swollen than the left side. A hard cord-like mass was palpated in the right cheek, extending from the zygomatic bone to the anterior edge of the mandibular angle. His temporomandibular joint is immobile but depleted of any primary joint problems. His mouth was tightly closed with a deep anterior overbite and overjet. However, the patient’s thumb valgus deformity is not obvious (Fig. 1).

### 2.3. Imaging examination

**Figure 2.**
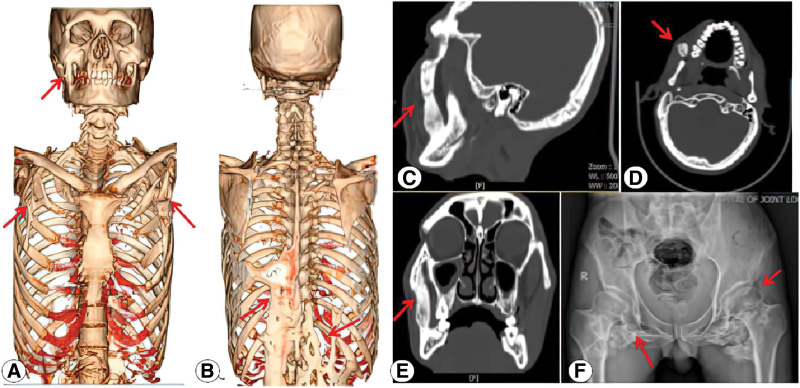
Three-dimensional reconstruction image of head, chest, and abdomen and X-ray image of the pelvis. (A) Ectopic ossification in masseter, scapular region; (B) Bony connections in the lower part of the spine; (C) sagittal view of ectopic ossification in masseter; (D) axial view of ectopic ossification in masseter; (E) coronal view of ectopic ossification in masseter; (F) high-density shadow around the bilateral femur and ilium.

Three-dimensional reconstruction of CT scan of the maxillofacial region showed enlargement of the right zygomatic bone, with an “arch-shaped” bone-like tissue located in the anterior border of masseter muscle, extending downward from the lower edge of the right zygomatic bone to the anterior edge of the mandibular angle. Spur-like irregular protrusions were observed in the mandibular angle. Three- dimensional reconstruction of CT scan of the chest and upper abdomen showed prominent ossifications across the chest, back, and waist, surrounding the thoracic cage, acromioclavicular joint, and vertebrae, resulting in bony bridge and bone fusion between affected bones. The X-ray film of the pelvis revealed a high-density shadow around the bilateral femur and ilium (Fig. 2).

### 2.4. The course of treatment

Under the patient’s strong desire to resolve his mouth-opening problem, the patient underwent the surgical resection of ossification in the masseter muscle and removal of the wisdom tooth via an intraoral incision under general anesthesia with fiberoptic nasotracheal intubation. Postoperative images showed that the high-density ossification image of masseter area had been removed. The mouth opening was about 1.5 cm after surgery and the patient was instructed to insist on mouth-opening exercises and take glucocorticoids and nonsteroidal anti-inflammatory medications after discharge. After follow-up for more than a year after surgery, the patient’s mouth opening is <1 cm, being slightly less than that immediately after surgery. Two years after surgery, the patient stated the recurrence of mouth restriction symptoms.

### 2.5. Postoperative pathology

**Figure 3.**
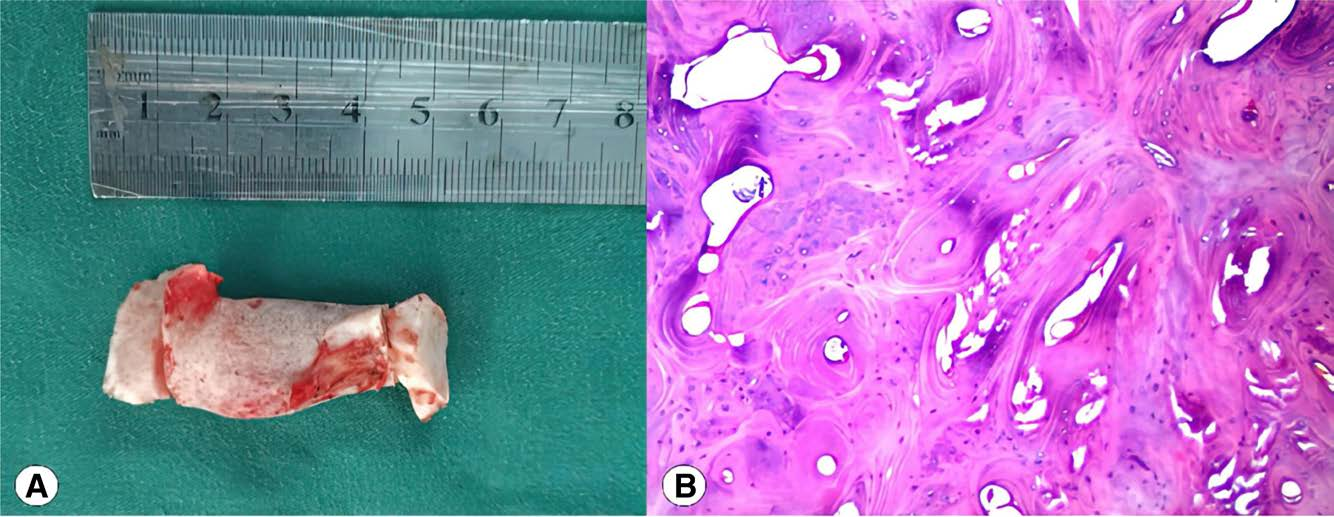
Histopathological examination. (A) Resected tissue specimens in masseter muscle; (B) histopathological examination of the resected tissue specimens confirmed mature bone tissue.

Histopathological examination of the resected tissue specimens confirmed mature bone tissue (Fig. 3).

### 2.6. ACVR1 gene analysis

**Figure 4.**
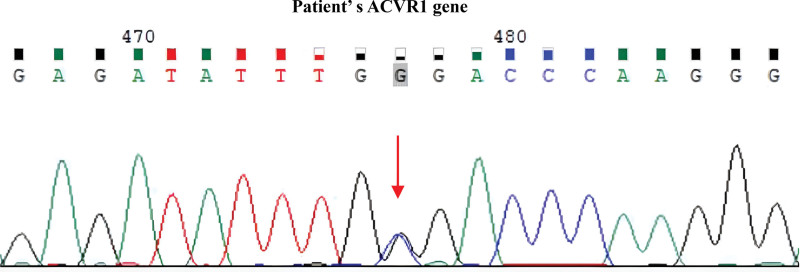
Electropherograms of the mutations found in ACVR1 gene: the new c.974G > C transition causing the p.G325A residue change.

The results of genetic testing showed the patient had a heterozygous molecule (c.974G > C, p.G325A) in ACVR1 (Fig. 4).

Combining the medical history, clinical manifestations, imaging features, genetic analysis, and pathological findings, the patient was diagnosed with FOP complicated with localized MO in masseter after trauma and infection.

## 3. Discussion

FOP is usually diagnosed based on clinical manifestation and imaging examination. Nevertheless, genetic analysis is also needed.^[3]^ Molecule (c.617G > A, p.R206H) in the ACVR1 gene have been found in most reported FOP patients, individuals with this classical molecule have an early onset of ossifications.^[4]^ The course of the disease is usually severe and leads to immobility at an early age.^[5]^ The first ectopic ossifications in this group of patients usually appear in childhood and are frequently manifested as spontaneous bony nodules.^[6]^ Almost all the patients noticed the congenital malformation of the big toe.^[7]^ The gradual emergence of ectopic ossification not only leads to immobility of all major joints but also respiratory dysfunction caused by severe scoliosis; most patients experience difficulty breathing in the third to fourth decade of life.^[8]^

Our patient with FOP is noteworthy because he carries a novel molecule in ACVR1(c.974G > C, p.G325A). His heterotopic ossification occurred in the middle-aged and symptoms were mild, his congenital deformity of thumb was not obvious. Therefore, it could be predicted that the amino acid changes p.G325A seem to be related to a milder phenotype with later onset of the disease and less influence on life quality than in patients with the classical molecule, but the ossification would be affected by exogenous factors such as trauma because under exogenous stimulations, the activity of ACVR1 would be further enhanced.^[9]^ The patients with FOP are more susceptible to stimuli, which might aggravate their symptoms of ectopic ossification.

Once the ossification involves the maxillofacial region, the most frequent symptom is trismus, so when a patient is unable to open their mouth, especially with a history of recent oral surgery, FOP or localized MO should be considered despite the rare incidence. Besides, when heterotopic ossification develops in the spine, some features of the radiographic findings in the spine and sacroiliac joints could be misinterpreted as ankylosing spondylitis. Some cases of FOP similar to ankylosing spondylitis have been reported.^[10]^ FOP should also be differentiated from other similar conditions, including progressive osseous heteroplasia, osteoma cutis, sarcoma, and desmoid tumor.^[11]^

Currently, therapeutic interventions are still supportive and preventive. If FOP is suspected, all elective procedures, including surgery, biopsies, intramuscular injections, and vaccinations, should be postponed until a definitive genetic diagnosis is confirmed.^[12]^ After definite diagnosis, the treatment of FOP is concentrated on drug therapy, injury prevention, and surgical treatment in accordance with FOP treatment guidelines.^[13]^

Medication is the first option for patients with FOP, while only a few clinically effective therapies for FOP are available. Standard therapies include glucocorticoid, nonsteroidal anti-inflammatory medications, cyclo-oxygenase-2 inhibitors, leukotriene inhibitors, and mast cell stabilizers.^[14]^ The effect of glucocorticoid on acute attack was emphasized, nonsteroidal anti-inflammatory medications, cyclo-oxygenase-2 inhibitors, leukotriene inhibitors, and mast cell stabilizers are effective in treating persistent discomfort and flare-ups.^[15,16]^ In 2023, Palovarotene has been approved by the FDA for the treatment of FOP in girls 8 years of age and boys 10 years of age and older, which is a selective retinoic acid receptor gamma agonist and intended to mediate interactions between receptors in the retinol signaling pathway, reducing the formation of new abnormal bones.^[17]^

Besides, drugs such as IL-1 inhibitors, tofacitinib, garetosmab, saracatinib, and rapamycin are being tested for FOP treatment.^[13]^ Ruby et al^[18]^ found that IL-1 inhibitors appear to decrease the frequency and severity of FOP flares, which may minimize the requirement for glucocorticoids during flares and enhance patient-reported well-being. As Tofacitinib recently was approved by FDA for polyarticular juvenile idiopathic arthritis, Irina et al^[19]^ suggested that Tofacitinib’s anti-inflammatory impact can control the flares of FOP and prevent or reduce the ossifications. Maja et al^[20]^ demonstrated that adult patients with FOP treated with garetosmab experienced a substantial and durable reduction in the formation of new ectopic bone lesions and episodes of soft tissue inflammation. Hino et al^[21]^ found that saracatinib effectively inhibits chondrogenic development and presented additional efficacy data in FOP mouse models. Rapamycin has been demonstrated to reduce trauma-induced and constitutively active ALK2-induced ectopic bone formation.^[22]^

In addition, the stimulating factors which may induce ossification should be actively removed in the treatment.^[23]^ In this case, the previous anti-infection and debridement for osteomyelitis did not treat the diseased tooth in time, which might result in a series of proliferative reactions and aggravate the ossification in masseter.

Doctors should avoid unnecessary surgical resection of heterotopic ossification. Surgical resection may be performed in cases of excessive pain, joint limitation, nerve compression, or severe impact on the patient’s quality of life,^[24]^ preferably in the mature stage of the disease, at this point the ossified area can be clearly demarcated from the surrounding skeletal muscle.^[25]^ After operation, patients should be advised to strengthen their physical exercise.^[2]^ However, the surgery still carries a significant risk for FOP patients, as it may stimulate inflammation, aggravate local ossification, and easy to relapse.^[26]^ In this case, the patient had a strong desire for surgery because ossification of masseter had severely affected eating. X-ray examination showed that the density of the affected lesion was as high as that of the adjacent cortical bone, suggesting that ossification was mature. After comprehensive consideration and informing the patient of the surgical risk, ossification of masseter muscle was resected, then the patients were instructed to carry out mouth opening training and take glucocorticoids and nonsteroidal anti-inflammatory medications.

Recently, gene therapy has also become a promising means to cure FOP. A recent study demonstrated that genetic removal of Hedgehog can abolish heterotopic ossification in mouse models.^[27]^

In the future, the research of FOP will further focus on inflammation, dysregulation of BMP signaling, and endochondral ossification.^[28]^ The long-term methods to treat or prevent FOP include blocking the activity of the ACVR1 receptor, redirecting the progenitor cells in the inflammatory environment away from adopting an osteogenic fate toward more of a soft tissue fate, inhibiting inflammatory triggers of FOP, and altering the conducive microenvironment to encourage the development of FOP symptoms.^[29]^

In conclusion, this case suggests that distinct genotypic differences will result in diverse clinical symptoms for FOP patients. For patients with FOP, local trauma should be avoided and possible stimulation such as the symptomatic tooth should be actively dealt with to prevent further aggravation of ossification.

## Author contributions

**Data curation:** Yian Guan.

**Writing—original draft:** Yian Guan.

**Conceptualization:** Dongyang Ma.

**Project administration:** Dongyang Ma.

**Writing—review and editing:** Dongyang Ma.
